# A Multidisciplinary Investigation of a Polycythemia Vera Cancer Cluster of Unknown Origin

**DOI:** 10.3390/ijerph7031139

**Published:** 2010-03-17

**Authors:** Vincent Seaman, Steve M Dearwent, Debra Gable, Brian Lewis, Susan Metcalf, Ken Orloff, Bruce Tierney, Jane Zhu, James Logue, David Marchetto, Stephen Ostroff, Ronald Hoffman, Mingjiang Xu, David Carey, Porat Erlich, Glenn Gerhard, Paul Roda, Joseph Iannuzzo, Robert Lewis, John Mellow, Linda Mulvihill, Zachary Myles, Manxia Wu, Arthur Frank, Carol Ann Gross-Davis, Judith Klotz, Adam Lynch, Joel Weissfeld, Rona Weinberg, Henry Cole

**Affiliations:** 1 Agency for Toxic Substances and Disease Registry, 4770 Buford Highway NE, Atlanta, GA, 30341, USA; E-Mails: sed7@cdc.gov (S.D.); dfg0@cdc.gov (D.G.); bkl9@cdc.gov (B.L.); swm1@cdc.gov (S.M.); keo1@cdc.gov (K.O.); bgt2@cdc.gov (B.T.); jcz8@cdc.gov (J.Z.); 2 Pennsylvania Department of Health, 7^th^ & Forster Streets, Harrisburg, PA 17120, USA; E-Mails: jlogue@state.pa.us (J.L.); dmarchetto@state.pa.us (D.M.); sostroff@state.pa.us (S.O.); 3 Mt. Sinai School of Medicine, One Gustave L. Levy Place, New York, NY 10029-6574, USA; E-Mails: ronald.hoffman@mssm.edu (R.H.); mingjiang.xu@mssm.edu (M.X.); 4 Geisinger Health System/Clinic, 100 N. Academy Ave, Danville, PA 17822, USA; E-Mails: djcarey@geisinger.edu (D.C.); pmerlich@geisinger.edu (P.E.); gsgerhard@geisinger.edu (G.G.); proda1@geisinger.edu (P.R.); 5 Pennsylvania Department of Environmental Protection, 2 Public Square, Wilkes-Barre, PA 18711, USA; E-Mails: jiannuzzo@state.pa.us (J.I.); robelewis@state.pa.us (R.L.); jmellow@state.pa.us (J.M.); 6 Centers for Disease Control and Prevention, National Program of Cancer Registries, 1600 Clifton, Rd NE, Atlanta, GA 30333, USA; E-Mails: lmulvihill@cdc.gov (L.M.); hkt4@cdc.gov (Z.M.); mwu@cdc.gov (M.W.); 7 Drexel University School of Public Health, 1505 Race Street, Bellet Building 13th Floor, Philadelphia, PA, 19102, USA; E-Mails: ALF13@drexel.edu (A.F.); CG48@drexel.edu (C.A.G); Judith.Klotz@comcast.net (J.K.); 8 UPMC Cancer Pavilion, 3rd Floor, 5150 Centre Avenue, Pittsburgh, PA 15232, USA; E-Mail: jwepid@pitt.edu; 9 New York Blood Center, 310 East 67th Street, 2-47B, New York, NY 10065, USA; E-Mail: rweinberg@nybloodcenter.org; 10 Henry S. Cole & Associates, 7611 S. Osborne Rd, Ste 201, Upper Marlboro, MD 20772, USA; E-Mail: hcole@hcole-environmental.com

**Keywords:** polycythemia vera, cancer cluster, environmental exposure, Pennsylvania

## Abstract

Cancer cluster investigations rarely receive significant public health resource allocations due to numerous inherent challenges and the limited success of past efforts. In 2008, a cluster of polycythemia vera, a rare blood cancer with unknown etiology, was identified in northeast Pennsylvania. A multidisciplinary group of federal and state agencies, academic institutions, and local healthcare providers subsequently developed a multifaceted research portfolio designed to better understand the cause of the cluster. This research agenda represents a unique and important opportunity to demonstrate that cancer cluster investigations can produce desirable public health and scientific outcomes when necessary resources are available.

## 1. Introduction

In 2004, four cases of a rare blood cancer called polycythemia vera (PV) were found in people living on the same rural road near the borough of Tamaqua in northeast Pennsylvania. This finding led the Pennsylvania Department of Health (PADOH) to review cancer cases reported to the Pennsylvania Cancer Registry from the three counties (Carbon, Luzerne, and Schuylkill) surrounding the Tamaqua area. PADOH found that the overall cancer rate in the tri-county area was similar to that in other parts of the state, but there were more PV cases than expected [1]. In October 2006, PADOH asked the Agency for Toxic Substances and Disease Registry (ATSDR) to help further study the patterns of PV in the tri-county area.

PV is a myeloproliferative neoplasm (MPN) of the bone marrow characterized by an overproduction of erythrocytes and often other blood cells. Previously known as myeloproliferative disorders (MPDs), the MPNs include essential thrombocytosis (ET), primary idiopathic myelofibrosis (IM), and chronic myelogenous leukemia (CML). PV has no known cause and is estimated to occur in 1 of every 100,000 people each year [2]. The median age of diagnosis for PV is approximately 60 years, and males account for slightly more than half (58%) of the cases [3]. In 2004, an acquired point mutation in the Janus-activated kinase-2 (JAK2) gene was discovered which occurs in >95% of all PV patients and approximately 50% of those with ET or IM, but not in other cancers [4]. The resultant JAK2 V617F mutation in the bone marrow stem cell confers a loss of auto-inhibitory control and an overproduction of the various cell-types. Although PV is not considered a hereditary disease, familial clustering of cases has been reported [5,6]. In addition, three recent studies [7–9] all demonstrated that the malignant clone containing the JAK2 mutation is strongly associated with a particular germ line haplotype encompassing the JAK2 gene. This haplotype is defined by specific single nucleotide polymorphisms (SNPs) which occur at frequencies of 5–41% in European populations. Before the discovery of the JAK2 mutation, PV was diagnosed using a complex series of clinical and laboratory parameters. In 2008, the World Health Organization (WHO) updated the existing 2001 diagnostic recommendations for MPNs by including the JAK2 mutation as a major diagnostic criterion for PV. This change resulted in a simplified and more straightforward diagnostic rubric using a disease-specific biomarker, and was used by ATSDR to validate cases in their investigation [10].

The goals of the ATSDR investigation were to: (1) locate all cases of PV in Carbon, Luzerne, and Schuylkill counties, (2) confirm the diagnosis of the PV cases using medical records and testing for the JAK2 mutation, and (3) describe the characteristics of the suspected PV cases. As a result of the investigation, ATSDR identified a statistically significant (*p < 0.001*) cluster of 15 PV cases at the nexus of Carbon, Luzerne, and Schuylkill counties [11]. As a group, the confirmed PV cases did not report any employment, leisure activities, or other factors that were different from those reported by participants who were found not to have PV. The investigation also found that nearly half of the confirmed PV cases had not been reported to the Pennsylvania Cancer Registry, and that a third of the participants reported to the registry during this period with a physician’s clinical diagnosis of PV did not satisfy the 2001 or 2008 WHO PV criteria. The identification of the PV cluster is important because it is the first reported for any MPN, a group of diseases with unknown etiology. The presence of numerous potential environmental exposures in the cluster area—and the absence of any other apparent cause—create the possibility that external factors contributed to the cluster. In addition, serious questions were raised about the completeness and homogeneity of PV and other MPN cases reported to cancer registries, resulting in part from the recent addition of the JAK2 mutation to the diagnosis criteria. In August 2008, ATSDR and PADOH convened an expert panel in Philadelphia, composed of medical researchers, environmental scientists, and public health professionals to review the findings and recommend future studies that would help explain the origin of the cluster and advance general knowledge about PV and other MPNs. This report summarizes the resultant research portfolio developed by ATSDR and the objective and rationale for each project.

## 2. Results and Discussion

The PV expert panel identified 4 major research areas: epidemiology, genetics/biomarkers, toxicology, and environmental analysis. Within these four areas, 13 separate projects were discussed. All the projects have the potential to provide new information about PV, but differ in scope. Some are specific to the PV cluster in the Tamaqua area while others involve broader geographic areas of Pennsylvania and/or other states or answer questions related to MPN etiology and reporting. ATSDR and PADOH prioritized the research proposals by evaluating the comments of panel members, the scope and potential benefits of each proposal, and the resources and time required to complete each project. Efforts were made to identify which institutions or agencies could most effectively address each project. As a result, 11 studies were selected for the PV research portfolio along with five additional non-research activities that address environmental data collection, community involvement and physician education (Table 1).

### 2.1. Epidemiologic Studies

#### 2.1.1. Case control study (Drexel University School of Public Health)

*Objective:* To identify risk factors associated with the development of PV in the cluster area.

*Methods/Rationale:* While the initial ATSDR study looked for commonalities among the individuals confirmed to have PV, it was not designed to identify a potential cause for the cluster. The expert panel believed it was important to formally assess possible risk factors for PV in the tri-county area. Such a study is challenging because both the cause for the MPNs and the time frame for development of disease after either a cancer-producing exposure or occurrence of the JAK2 mutation are unknown. The study will focus on a variety of external causes, especially those related to the environment, and hereditary or genetic influences. Persons with confirmed PV from the original ATSDR study will be enrolled, as well as those more recently identified, and will have appropriate non-ill control groups. The specific methods for conducting the case-control study are currently in development. The study is expected to be multiyear and to have an environmental sampling component.

#### 2.1.2. Comparative epidemiologic study (University of Pittsburgh Cancer Institute)

*Objective*: To explore the patterns of PV and other MPNs in another area of Pennsylvania with similar characteristics to the PV cluster area.

*Methods/Rationale:* A number of problems were identified in the initial ATSDR study regarding the accuracy of PV reports to the cancer registry. Many reported cases were found not to have the disease, and a number of confirmed cases that had not been reported to the registry during the time period of interest were found. These problems are unlikely to be unique to the tri-county area, and make it difficult to compare data from that area to those found in other parts of Pennsylvania or elsewhere in the country. It is possible that other locations also have excess rates of PV and MPNs, especially those that have similar characteristics to the tri-county area of northeast PA. If they do, it might help identify possible causes, and if they do not, it might help focus investigations in the cluster area on unique features of that location. The investigators will reproduce the original ATSDR study in another multi-county area in Pennsylvania that is largely rural, has a similar population size and demographics, and shares many other features with the cluster location. The primary task is to locate all PV cases diagnosed from 2001 to 2007 in the selected area, including those not reported to the Pennsylvania Cancer Registry, and verify the diagnoses using 2008 WHO criteria. Genetic testing will be offered, if necessary, to confirm the MPN diagnosis.

#### 2.1.3. PV and MPN surveillance (PADOH)

*Objectives*: To (1) continue surveillance of PV and other MPNs reported to the Pennsylvania Cancer Registry in both the cluster area and in other areas of Pennsylvania, (2) verify the diagnosis and evaluate the reporting frequency of newly diagnosed cases, and (3) provide logistical and technical assistance to other research partners working in Pennsylvania.

Methods/Rationale:

1. PV Surveillance—PADOH will assess and classify cases of PV reported to the cancer registry from the tri-county area since the joint PADOH/ATSDR survey was conducted in 2005. Other counties in Pennsylvania reporting high rates of PV compared to the state average will also be evaluated. The PV diagnosis will be confirmed using 2008 WHO guidelines by reviewing medical records and offering a JAK2 analysis, if needed. Non-reported cases in the tri-county area will be identified through regional hematologists/oncologists.
2. MPN Surveillance—The MPNs represent an inter-related series of diseases that may have a common origin, and CML, ET, and IM have not yet been evaluated in the tri-county area. Since there is an apparent excess of PV in the area, the other MPNs may be similarly elevated. Persons identified in the Pennsylvania Cancer Registry since 2001 with any of these three diseases will be assessed through questionnaires and will be offered genetic testing, if necessary, for validation of their diagnosis. Data in the registry related to these disorders from other areas of the Commonwealth will also be reviewed.
3. Logistical/Technical Support—PADOH will assist other research partners by providing community outreach and support, local clinical laboratory services (where available), and logistical and technical assistance. This will include coordinating with community leaders; providing educational and informational materials to local residents; and offering clinical services to PV research partners, including blood draws, specimen storage and transfer to outside laboratories. PADOH will also assist research partners with data needs with respect to the cancer registry and other state databases.

#### 2.1.4. Patterns of MPN diagnosis, reporting, and care (Geisinger Clinic)

*Objective*: To determine the patterns of diagnosis, treatment and reporting for MPN patients by physicians in northeast Pennsylvania.

*Methods/Rationale:* Health records of MPN patients residing in Carbon, Luzerne and Schuylkill Counties who are being seen by Geisinger physicians will be scanned for the presence of an MPN diagnosis. The percentage of these patients that are tested and that carry a known MPN-related mutation will be determined. The primary treatment modalities and the registry reporting status of each patient will be documented. Education and/or information about new diagnostic tests and guidelines for MPNs will be provided to physicians of MPN patients. The results will be of value in future tumor registry studies and supplement similar work by PADOH (see 2.1.3.).

#### 2.1.5. Prospective study of JAK2-positive persons without MPN or with mild MPN in the tri-county area (Geisinger Clinic)

*Objectives*: To fully characterize subjects who carry the JAK2 mutation but either do not meet the 2008 WHO diagnostic criteria for a MPN, or meet the diagnostic criteria but have mild disease (*i.e.*, not requiring therapy), and to follow the natural progression of the disease to a clinically active MPN requiring therapy.

*Methods/Rationale:* Screening for the JAK2 mutation was offered to cluster area residents as a public service in late 2009. A number of JAK2-positive individuals were identified who were asymptomatic or had minimal evidence of disease. These individuals have been asked to participate in this study. It is anticipated that no more than 20 individuals will be eligible. Each participant will require an initial consultation and a thorough medical history and physical exam. Periodic assessments, including a complete blood count and quantitative JAK2 assay, will be performed at least every 6 months to monitor progression of the mutation burden. Enrolled subjects who are found to have evidence of PV or another MPN requiring treatment will no longer be eligible for participation in this study. They will be treated according to standard procedures for the clinical management of these conditions. They may also be invited to participate in other MPN-related clinical trials, as appropriate.

#### 2.1.6. Enhancement of physician reporting of hematologic cancers, including PV, in the CDC supported National Program of Cancer Registries (Centers for Disease Control and Prevention)

*Objective*: To evaluate and enhance the reporting of PV and other hematologic cancers to central cancer registries.

*Methods/Rationale:* The validity of data collected and reported by central cancer registries depends, in part, upon the completeness and accuracy of data reporting. While hospitals routinely report cancer cases, reporting from outpatient locations varies widely. Thus, cancers such as MPNs, which are diagnosed in an outpatient setting, are often under-reported. Cancer registries are not equipped to validate or confirm diagnoses, and rely upon the medical practitioner to accurately identify reportable cases. Changes in the diagnostic definition of a particular cancer created by the discovery of new techniques, such as biomolecular or genetic markers, are reflected in cancer registry data only to the extent they are adopted by physicians. Because these changes in medical practice take place over a period of time and through a consensus process within the medical community, registry data during this transition includes cases diagnosed using different disease definitions. This lack of homogeneity may affect measurement of disease trends. The CDC’s National Program of Cancer Registries will fund a select number of cancer registries to improve and enhance reporting of PV and other hematopoietic diseases diagnosed in physician offices. The work will focus on evaluating current MPN diagnosis and reporting practices, increasing physician awareness of reporting requirements and processes, and building infrastructure at the physician and state health department level for electronic reporting of hematologic cancers from hematology practices.

#### 2.1.7. Geospatial analysis of MPN residences (ATSDR)

*Objective:* To continue the geospatial analysis of the PV cluster, assess the impact of newly found cases and evaluate current and historic residences of PV cases, other MPN cases, and asymptomatic JAK2-positive individuals with respect to potential environmental risk factors identified by the on-going work.

*Methods/Rationale:* Newly-found cases of PV and other MPNs, along with non-MPN JAK2-positive individuals identified in the screening project (see 2.2.2.) will be added to the existing database. The results of the environmental testing and other relevant studies will be used to evaluate associations between PV cases and potential exposure sources, assess specific agents and exposure pathways, and conduct comparative analyses with other potential cluster areas. This work will involve the use of standard geospatial tools, space-time analyses, and non-traditional methods such as those used in the initial cluster identification [11].

### 2.2. Genetic and Biomarker Studies

#### 2.2.1. JAK2 screening in the cluster area (ATSDR)

*Objective:* To conduct JAK2 screening as a community service in and around the PV cluster area to identify individuals carrying the JAK2 mutation.

*Methods/Rationale:* Residents who have lived in the tri-county study area for at least one year were eligible to participate in the screening. Blood-draw clinics were established in three locations in and around the cluster area during two separate screening events in August and October of 2009. Specimens were shipped overnight to the molecular diagnostic laboratory at Mt. Sinai School of Medicine for JAK2 analysis. Specimens were first screened qualitatively for the JAK2 mutation using a one-step amplification refractory mutation system polymerase chain reaction (PCR) method. The assay has a sensitivity of 0.01% of JAK2 allele in a wild-type background and a specificity of >99.5%. All positives were re-tested using a real-time PCR quantitative assay to confirm the qualitative result and determine the JAK2 allele burden. Individuals who tested positive for the JAK2 mutation were invited to participate in a follow-up study which will monitor their JAK2 burden and hematologic status every 6 months for a three year period (see section 2.1.5.).

#### 2.2.2. JAK2 prevalence study (Geisinger Clinic)

*Objectives:* To (1) systematically evaluate the prevalence of the JAK2 mutation in healthy individuals from the cluster area and from parts of Pennsylvania outside the tri-county area, and (2) genotype DNA from a subset of the patients for two JAK2 susceptibility SNPs.

*Methods/Rationale:* Geisinger Clinic is part of the Geisinger Health System, which provides primary care to residents of over 40 counties in central and eastern Pennsylvania, with over 2.6 million people living in the primary service area. Geisinger has implemented a robust electronic health record system (EPIC^®^) which manages all aspects of patient care and treatment, and a biobank (MyCode^®^) with over 23,000 consented participants and 56,000 blood specimens available for research purposes. The EPIC^®^ and MyCode^®^ systems will be used to generate a random sample of 2,500 specimens from within the cluster area and 6,000 specimens from outside the tri-county area which are age- and gender-adjusted to the cluster area. Demographic and health data will be available for each specimen donor, including the results of any additional laboratory tests that have been performed (e.g., complete blood count, hematocrit, hemoglobin, lactate dehydrogenase, erythropoietin).

1. *JAK2 Prevalence.* The JAK2 V617F TaqMan RT-PCR Assay (Applied Biosystems, Carlsbad, CA) will be used to identify the JAK2 mutation in DNA extracted from the selected blood specimens. The method will be validated against the nested allele-specific PCR assay used in the JAK2 screening project (see section 2.2.1.), and all specimens testing positive will be sent to the Mt. Sinai molecular diagnostic laboratory for validation and quantification of the JAK2 mutation.

2. *JAK2 Susceptibility Haplotype.* Three recent studies [7–9] all demonstrated that the malignant clone containing the JAK2 mutation was strongly associated with a particular germ line haplotype that encompassed the JAK2 gene and was defined by SNPs. This susceptibility haplotype can be defined by the SNPs rs12343867 (risk allele C; non-risk T) and a tagged SNP rs12340895 (risk allele G; non-risk C). The reported frequencies of these SNPs/haplotypes vary slightly among European derived populations, with homozygous minor allele frequencies of 5% to 7% and heterozygous frequencies of 38% to 41%. Genotyping 2500 cluster-area specimens and 2500 controls (ensuring at least 100 homozygotes in each group) provides sufficient power (>80%; alpha = 0.05) to detect a difference in homozygous frequencies of ~6% to ~8%, and heterozygous difference from ~40% to ~44%. The frequency of the susceptibility haplotype and association of the haplotype with presence of the somatic JAK2 mutation will be compared in the cluster area and control area specimens.

#### 2.2.3. Gene profiling study (Mt. Sinai School of Medicine)

*Objective:* To analyze PV cluster-area patient specimens and a control population from other geographic areas by gene profiling analyses and Genome-Wide SNP Association Platform (SNAP) arrays to detect genetic precursors to MPNs and/or unique signatures specific to the MPN population in the Tamaqua area.

*Methods/Rationale:* Total RNA and genomic DNA will be prepared from granulocytes isolated from PV patients from the study area and PV patients geographically distant from the study area. The Affymetrix Human Genome U133 plus array will be used with normalized data obtained from GeneChip® Operating Software analysis of the Affymetrix Human Genome U133 plus chips. A Significance Analysis of Microarrays using the Institute for Genomic Research MultiExperiment Viewer software package will be performed to determine probes that show the highest differential expression between study area and non-study area PV samples. The lists of up- or down-regulated genes in study area and non-study area PV specimens will be determined and p-values calculated.

#### 2.2.4. Tissue bank for cluster-area MPN patients (MPD-Research Consortium, New York)

*Objective:* To collect and store tissue samples from MPN patients in the cluster area.

*Methods/Rationale:* The MPD-Research Consortium (MPD-RC) has established an MPD tissue bank at the New York City Blood Center. The MPD-RC will provide support for patient recruitment and evaluation; tissue specimen collection, storage, and shipping; and other activities as specified by the tissue bank protocol. Tissue specimens collected from up to 140 living patients and 10 post-mortem MPN patients will include bone marrow aspirate, bone marrow biopsy, toenail clippings, spleen cells, and blood. Other tissues, and tissues from patients without an established MPN diagnosis, may be collected if deemed appropriate by the tissue bank or the attending physician. The collected samples will be used to conduct laboratory investigations to help define mechanisms involved in the pathophysiology of these disorders, possible causes of the cluster, and potential treatments.

### 2.3. Toxicology Studies

#### 2.3.1. Bone marrow toxicological assay (Mt. Sinai School of Medicine)

*Objective:* To develop a physiological *in vitro* assay for stem cell-derived progenitor cells to evaluate potential toxins present in the cluster area that may cause PV.

*Methods/Rationale:* An *in vitro* cell culture system that captures essential features of the *in vivo* erythroid micronucleus genotoxicity assay will be used to identify environmental agents in the study area that might increase hematopoietic DNA damage. Ten pure toxic compounds will be selected from the potential contaminants in the study area based on their ability to cause DNA damage in mammalian cells and the likelihood of past human exposure in the cluster area. The effects of agents which induce or inhibit metabolic activation/detoxification processes (*i.e.*, P450 enzymes) will also be evaluated. Three known genotoxicants that test positive in the *in vivo* erythroid micronucleus assay will serve as positive controls for this study, specifically BCNU (1,3-bis[2-chloroethyl]-1-nitrosourea), MNNG (N-methyl-N*-nitro-N-nitrosoguanidine), and MMS (methyl-methane sulfonate) [12]. The selected compounds or controls will be added to normal human stem/progenitor cells for 1h, 4h, 12h or 24h at various concentrations. The Comet assay and micronucleus test will be used to screen for DNA damage caused by environmental mutagens. These assays will be repeated on 10–15 normal donors for each toxic compound. Archived specimens from PV patients in the study area will be evaluated for DNA irregularities in the *in vitro* culture caused by selected environmental agents.

### 2.4. Environmental Studies

#### 2.4.1. Environmental exposure assessment data inventory (ATSDR)

*Objective:* To compile an integrated database of all federal, state, and other relevant data and documents pertaining to hazardous waste sites, human exposures, environmental testing, and industries or businesses that release toxic materials in the identified cluster area.

*Methods/Rationale:* ATSDR will create a comprehensive database that combines the extensive collection of data and other geospatial resources from the tri-county and identified cluster area into a useful research tool, and will allow researchers to access data by site, substance, exposure pathway, and time period. This data warehouse will include:

- Environmental data, including types and locations of industries and hazardous waste sites; land use and cover; water use and distribution; air emission inventories and sources of air pollution; waste management information; and environmental sampling information for chemicals and radionuclides released into air, water, soil, and biota.
- Topographic data, including roads, water features, and base maps that define legal boundaries and serve as reference points.
- Demographic data, including the age, race, sex, education, and income of the population in the tri-county area.
- Health outcome data, including PV and MPN incidence and surveillance data, JAK2 screening and prevalence data, and other health conditions identified during the PV investigations.

While the data warehouse is designed primarily to study potential relationships between environmental exposures, PV and the JAK2 mutation in selected portions of the tri-county area, the information will also be useful for the study of other diseases in the tri-county area and as a reference for other areas with similar problems.

#### 2.4.2. Environmental testing (Pennsylvania Department of Environmental Protection)

*Objectives:* To obtain comprehensive environmental data regarding (1) water outflows from the McAdoo Superfund site; (2) indoor and outdoor contaminants at current and past residences of confirmed PV patients and other residential properties in the cluster area; and (3) water monitoring at three waste coal cogeneration plants in the study area.

Methods/Rationale:

1. *McAdoo Outflow Testing.* Water and sediment samples will be collected from a minimum of 20 different locations at the McAdoo Associates site to provide data that describes the surface water and groundwater outflow to the south. The locations will be sampled for volatile organic compounds (VOCs), semi-volatile organic compounds (SVOCs), heavy metals, polychlorinated biphenyls (PCBs) and radioactivity at least two times over the period of 1 year to reflect seasonal variations.
2. *PV Residence Testing*. Drinking water quality, soil, indoor dust, and residential radon levels will be tested at the current and past residences of confirmed PV patients and at a number of other sites in the cluster area. Samples will be tested for VOCs, SVOCs, heavy metals, PCBs, herbicides, pesticides and radioactivity.
3. *CoGeneration Water Monitoring Testing*. Water samples will be collected from various locations at three waste coal CoGeneration plants in the study area. The selected sites will be sampled for VOCs, SVOCs, heavy metals, PCBs, radioactivity (gross alpha & beta), and other water chemistry parameters (e.g., nitrate/nitrite, fluoride, sulfates, alkalinity) on two separate occasions to reflect seasonal variations.

### 2.5. Community Involvement/Education Activities

#### 2.5.1. Community Action Committee (Henry S. Cole & Associates)

*Objective:* To form a Community Action Committee which will represent the cluster-area communities in communications with the various environmental, public health, and MPN research groups and organizations.

*Methods/Rationale:* The Community Action Committee will be built around an existing core group of local residents with the intension of giving a voice to the various community interests. The committee will conduct regular meetings; produce reports, minutes and other documents to assist the community in understanding technical information on relevant environmental health issues. The committee will also provide informational support for MPN patients and their families, including rosters of area hematologists and other specialists, access to treatment and clinical trials, and an interface with government organizations on key patient issues.

#### 2.5.2. Physician education (Geisinger Clinic)

*Objective:* To provide training and education, including CME-eligible lectures, to physicians in northeast Pennsylvania on diagnosis, treatment, and reporting of PV and other MPNs.

*Methods/Rationale:* The educational programs will occur on a bi-monthly basis at local hospitals (*i.e.*, grand rounds) and other venues convenient for local practitioners. The materials developed will improve reporting of hematological cancers and raise awareness of the increased PV incidence in the cluster area. The educational programs will be available to representatives from other medical groups in Pennsylvania and other states and will be presented at a minimum of one regional and one national conference within the two year project period.

### 2.6. Oversight and Management (ATSDR)

*Objective:* To provide oversight and technical support for all PV research projects.

*Methods/Rationale:* ATSDR will encourage and initiate collaboration between partners, provide technical details or data required by partners to perform their work, and coordinate meetings with community representatives.

## 3. Conclusions

Hundreds of suspected cancer clusters are reported to state and federal public health agencies each year, the majority of which are evaluated and resolved quickly because they include a wide range of cancers, involve a small number of reported cases, lack potential exposure source(s) or exposure data, or because the occurrence of cases is within a normal range for the country [13]. For the few clusters that are investigated, the results are nearly always disappointing to the community since, even if the cluster can be verified, the cause usually cannot. Accordingly, state and federal public health agencies apply their limited resources to competing or more urgent priorities with a greater chance for a meaningful outcome. The current cluster investigation is thus unique in that it has the full support of both federal (ATSDR) and state (PADOH) public health agencies. Moreover, instead of the single potential exposure source commonly associated with cancer clusters, the PV cluster requires assessment of myriad potential environmental influences–hazardous waste sites, industrial emissions and waste, and naturally-occurring radiation sources–in addition to possible inherited genetic risk factors. Finally, and most importantly, the investigation is being supported by significant financial and technical resources. This support is rare in cancer cluster studies, and allows for a comprehensive research portfolio that will, regardless of whether the specific cause of the cluster can be determined, result in benefits to local residents, MPN and cancer patients elsewhere, and the medical research community. These benefits include the community JAK2 screening and follow-up study, comprehensive testing of residential properties, the MPN patient support group, ready access for local MPN patients to clinical trials, and increased local physician awareness of MPN diagnosis and treatment. Also, even if environmental agents cannot be linked to the PV cluster, the enhanced evaluations may result in additional clean-up or regulatory actions and improvements in local environmental quality. More far-reaching impacts are the evaluation of other potential high-rate areas of PV in Pennsylvania; improvements in the diagnosis and reporting of MPNs to national cancer registries (and thus a better understanding of the true incidence of these cancers); the determination of the JAK2 mutation prevalence in the general population; a better understanding of the etiology and inter-relationship of MPNs, along with associated genetic risk factors, which may lead to improved treatments and prevention strategies; improved geospatial tools and methods for cancer cluster analysis; and the environmental exposure data inventory, which will be a valuable resource to the current PV research groups and others studying similar environmental cancer links.

This multifaceted research partnership is a unique and important opportunity to demonstrate that cancer cluster investigations can produce desirable public health and scientific outcomes if the necessary resources are available.

## Figures and Tables

**Table 1.** Polycythemia vera research projects, duration, and partners.

**Project**	**Period**	**Partner**
**Epidemiologic Studies**		
Case-control study in the cluster area	2 years	Drexel University School of Public Health
Comparative epidemiologic study in western Pennsylvania	2 years	University of Pittsburgh Cancer Institute
PV and MPN surveillance in Pennsylvania	3 years	Pennsylvania Department of Health (PADOH)
Patterns of MPN diagnosis, reporting, and care	3 years	Geisinger Clinic
Prospective study of JAK2-positive non-MPN in the tri-county area	3 years	Geisinger Clinic
Enhancement of physician reporting of hematologic cancers in the CDC National Program of Cancer Registries	3 years	Centers for Disease Control and Prevention (CDC)
Geospatial analysis of MPN residences	3 years	ATSDR
**Genetic and Biomarker Studies**		
Gene profiling study	3 years	Mt. Sinai School of Medicine
JAK2 screening in the cluster area	1 year	ATSDR
JAK2 prevalence study	1 year	Geisinger Clinic
Tissue bank for cluster-area MPN patients	3 years	MPD-Research Consortium
**Toxicology Studies**		
Bone marrow toxicological assay	3 years	Mt. Sinai School of Medicine
**Environmental Studies**		
Environmental exposure assessment and data inventory	2 years	ATSDR
McAdoo superfund site outflow testing	2 years	Pennsylvania Department of Environmental Protection (PADEP)
Residential testing	2 years	
Cogeneration water monitoring testing	2 years	
**Community Involvement/Education Activities**		
Community action committee (CAC)	2 years	Henry S. Cole & Associates
Physician education	3 years	Geisinger Clinic
